# Creatine monohydrate supplementation changes total body water and DXA lean mass estimates in female collegiate dancers

**DOI:** 10.1080/15502783.2023.2193556

**Published:** 2023-03-24

**Authors:** Samantha J. Brooks, Darren G. Candow, Annie J. Roe, Bethaney D. Fehrenkamp, Victoria C. Wilk, Joshua P. Bailey, Lukas Krumpl, Ann F. Brown

**Affiliations:** a University of Idaho, College of Education, Health & Human Sciences, Department of Movement Sciences, Moscow, ID, USA; b University of Regina, Aging Muscle & Bone Laboratory, Faculty of Kinesiology & Healthy Studies, Regina, SK, Canada; c University of Idaho, College of Agricultural and Life Sciences, Department of Family and Consumer Sciences, Moscow, ID, USA; d University of Idaho, Idaho WWAMI Medical Education Program, Moscow, ID, USA

**Keywords:** Creatine, body composition, cognition, supplementation, female collegiate dancer

## Abstract

Collegiate dance is unique because it requires athletic and academic performance; therefore, optimizing physical and mental function is crucial. Research among athletic populations demonstrate improvements in body composition, performance, and cognition following creatine monohydrate (CR) supplementation, yet dancers have not been investigated. The purpose of this study was to determine the effects of CR supplementation on body composition, performance, and cognitive function in female collegiate dancers. Participants were randomized to CR (CR; *n* = 7; 0.1 g·kg −1·day −1 CM +0.1 g·kg −1·day −1 corn-starch maltodextrin) or placebo (PL; *n* = 6; 0.2 g·kg −1·day −1 corn-starch maltodextrin) for 42 days. Pre- and post-testing included body composition, total body water (TBW), Depression, Anxiety and Stress Scale, Diet History Questionnaire, the National Institute of Health Toolbox fluid cognition battery and isokinetic strength, vertical jump, medicine ball throw, and Wingate anaerobic power test. CR demonstrated a significant increase in TBW (pre, 32.2 ± 3.5 kg; post, 32.7 ± 3.6 kg; *p* = 0.024) and lean mass (LM; pre, 39.8 ± 3.6 kg; post, 41.5 ± 4.5 kg; *p* = 0.020). CR supplementation may be an effective strategy to increase TBW and estimates of LM in female collegiate dancers. Although this may optimize aesthetics, larger samples sizes with resistance training are needed to determine if CR supplementation increases muscle mass and translates to improved performance.

## 1. Introduction

Exogenous creatine monohydrate (CR) supplementation is a safe and effective ergogenic aid commonly ingested by athletes to increase lean mass (LM), improve muscular power and strength, and promote skeletal muscle adaptations [1]. Although adenosine diphosphate can be re-phosphorylated through both aerobic and anaerobic processes, exogenous CR is most effective for the re-phosphorylation of adenosine diphosphate during high-intensity and short-duration exercises sessions such as resistance training [2]. Subsequently, the vast majority of research involving CR supplementation has examined its efficacy when combined with resistance training. However, there is some evidence that CR supplementation alone [3]. Mechanistically, CR supplementation has been shown to increase cellular hydration status [4] which may stimulate variables involved in the muscle protein synthetic process, such as satellite cells, growth factors, and protein kinases downstream in the mTOR pathway [5] Furthermore, creatine demonstrates anti-inflammatory and anti-catabolic effects in muscle [6] and increases calcium-reuptake into the sarcoplasmic reticulum and glycogen resynthesis [7] which may create a favorable environment for greater LM and strength over time.

In addition to LM and muscle performance improvements, CR supplementation has been shown to possibly increase measures of cognitive function by increasing serotonin levels in the brain [8]. Some research suggests CR content in the pre-frontal cortex has been inversely associated with depressive symptoms, in healthy adults, due to its ability to cross the blood-brain barrier thereby augmenting brain CR with the use of CR supplementation; however, these results are mixed [9,10]. In addition, following 8 weeks of CR supplementation, Hellem et al. [11] showed decreases in anxiety symptoms in individuals with methamphetamine dependence [11], albeit these implications in a healthy population are undetermined.

The vast majority of research involving CR supplementation has focused on young healthy males, with minimal attention given to young females [2]. From a generalizability perspective, it is important to investigate the efficiency of CR supplementation in young healthy females who participate in a sport that involves both aerobic and anaerobic energy systems (i.e. collegiate dancers). In the collegiate environment, dancers must balance rigorous academic demands including course work, assignments, and exams, as well as the physical demands of dance training, rehearsals and performances. Additionally, as compared to other collegiate athletes, dancers do not have the same nutrition and training supports because they are housed in an academic department and are not part of athletics in a university setting. Due to the minimal resources and extreme time commitment in collegiate dance, previous literature has investigated ways to support body composition and performance through minimally invasive dietary interventions [12–16]. For example, collegiate dancers’ dietary intake is typically suboptimal and includes predominately highly processed convenience foods [14]. Additionally, their caloric intake (2040 ± 710 kcal·day **−1**) is often below the recommendations for extended bouts of low-intensity exercise, and their dietary protein is below the recommended daily allowance of 0.8 g·kg **−1** [14]. Recent research suggests protein consumption ranging from 1.6 to 2.2 g·kg **−1** and ≥2.3 g·kg **−1** ·day **−1** may lead to improvements in body composition index (BCI), specifically increasing fat-free mass (FFM) and decreasing fat mass (FM) [13]. Since collegiate dancers’ body composition is more reflective of a sedentary population with FM > 30%, improvements in BCI may elicit changes in collegiate dancers’ overall health [16].

CR supplementation in dancers has not been investigated. CR supplementation may elicit numerous benefits unique to the collegiate dancer since their environment poses distinct physical and academic demands. The collegiate eating environment and lack of strength and conditioning programming make it difficult for collegiate dancers to obtain an optimal body composition. Additionally, collegiate dancers often experience mental fatigue due to balancing academic and performance schedules longitudinally (i.e. nine months per year for an average of four years). Therefore, CR supplementation may be a novel solution to improve body composition, increase muscular strength and power, and improve cognitive function in collegiate dancers. Thus, the purpose of this study was to determine the effects of a 6-week CR supplementation intervention on body composition, anaerobic fitness, and cognition in female collegiate dancers. It was hypothesized that CR supplementation would result in increases in LM, anaerobic power, and cognitive function compared to placebo.

## 2. Materials and methods

The study was a randomized, double-blind, placebo-controlled intervention. The intervention included 6 weeks of dietary supplementation in addition to laboratory and performance familiarization for pre- and post-testing. A convenience sample of participants included degree majors and degree minors in the dance program at the University of Idaho. Exclusion criteria included the use of medications that could affect muscle biology (i.e. corticosteroids), ingestion of CR monohydrate or dietary supplements containing CR ≤4 weeks prior to the start of supplementation, preexisting kidney or liver abnormalities, or currently participating in resistance training.

**Figure 1.**
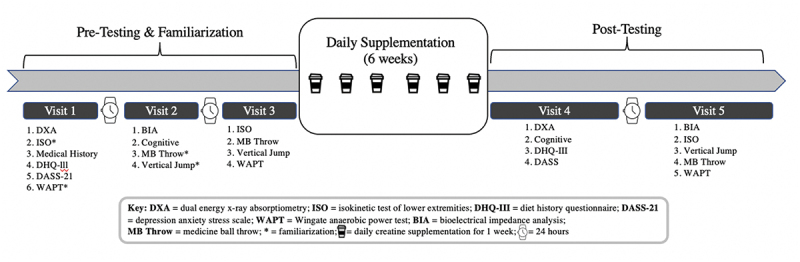
Schematic of Study Design.

Familiarization, pre-testing, and post-testing spanned 5 visits where the participants reported to the Human Performance Laboratory (HPL) at the University of Idaho (Figure 1.) Each individual participant was scheduled at the same time of day for pre- and post-testing between the hours of 0600 and 0900. Participants arrived to the HPL having fasted of food, caffeine, and alcohol for at least 12 hours and abstained from exercise outside of regular dance training for at least 24 hours. Participants were instructed to drink water ad libitum the day prior to pre- and post-testing and consume 8 ounces of water upon waking the day of testing in the HPL. At the beginning of visit 1, participants read and signed an informed consent explaining the procedures and potential risks and benefits of participation. Prior to visit 2, participants were matched based on body mass (kg), hours of dance training per week, meat intake (habitual CR intake), and menstrual cycle phase (follicular or luteal) and then randomized into either the CR or Placebo (PL) group. No differences in dietary intake were observed between groups. Participants in each group consumed their respective supplement with 0.24 L of water daily for 42 consecutive days (6 weeks, half of the academic semester) and were asked to maintain their regular dance training and habitual diet. Following the last day of supplementation, participants reported to the HPL to perform post-testing assessments. Details of each assessment are described in detail below. Upon completion of the study, participants were provided compensation for their time and were provided with their individual results.

### 2.1. Supplementation

Following pre-testing, participants were separated into two groups: CR (Creapure® AlzChem, Trostberg GmbH, Germany) and Placebo (PL; Globe® Plus 10 DE Maltodextrin, Univar Canada). CR consumed CR (0.1 g·kg **−1** ·day **−1** CR +0.1 g·kg **−1** ·day **−1** corn-starch maltodextrin) [17] and PL consumed only maltodextrin (0.2 g·kg **−1** ·day **−1** corn-starch maltodextrin). Both products were similar in taste, color (white), texture, and appearance, and were in powder form. Each supplement was reconstituted with 0.24 L of water for consumption. The purity of Creapure® was established at >99.9% by independent laboratory testing (The Cary Company, Addison, IL., USA). Participants visited the HPL between 1200 and 1400 hours during weekdays (Monday-Friday). On Friday, participants were given two doses of their supplement in a travel container to take home for consumption between 1200 and 1400 hours on the weekends. Participants consumed their respective supplements for 42 consecutive days. Photo and video evidence of supplement consumption was captured by participants and sent electronically to researchers for compliance assurance on weekend days. Participant supplementation compliance of >80% was required for inclusion in data analyses (i.e. >34 out of 42 days).

### 2.2. Body composition

Height and body mass were assessed prior to a dual energy X-ray absorptiometry (DXA) scan (Hologic DXA Scanner, Hologic Horizon^TM^; Danbury, CT). One noninvasive whole-body scan, anteroposterior view of the total body lying supine was performed. Testing was completed according to the manufacturer’s instructions and specifications. Results were analyzed with APEX software, version 4.5.2.1 (Hologic Inc.). The quality analysis for the densitometer was conducted daily using a standard aluminum spine block (Hologic Phantom) provided by the manufacturer. Measurements of the phantom fell within the manufacturer’s precision standard with a coefficient of variation <0.5%. Test-retest interclass coefficient of variation (CV; %) using DXA for LM (kg) and FM (kg) was 1.1% and 0.69%, respectively. The minimum detectable difference using DXA for LM (kg) and FM (kg) were 0.27 and 0.21 respectively. Total body water (TBW) was assessed via InBody scan (In-Body 270). Participants removed their socks and had their hands and feet wiped clean prior to standing on the InBody platform and grabbing the handles. Participants stood on the testing platform while holding hand electrodes for approximately 45 seconds. The minimal detectable difference using InBody for TBW (kg) was 0.19.

### 2.3. Questionnaires

Medical history questionnaire, Diet History Questionnaire (DHQ-III), Depression, Anxiety and Stress Scale (DASS): The medical history questionnaire inquired participants about exercise and dance training hours, previous injuries and surgeries, menstrual history, birth control use, and dietary preferences. The DHQ-III (https://epi.grants.cancer.gov/dhq3/) is an online food frequency questionnaire for adults of at least 19 years of age and is widely utilized by researchers to assess food and dietary supplement intake using 135 food and beverage line-items and 26 dietary supplement questions. Each line-item asked participants to record the frequency of intake and portion size of each item in the past 30 days. The DASS (Lovibond et al.,1995) is a set of 21 self-report questions designed to measure the emotional states of depression, anxiety, and stress and is a valid and reliable tool to assess changes in these mental health measures.

### 2.4. Cognitive assessment

Subjects completed the National Institute of Health Toolbox (NIHTB) fluid cognition battery, which included 5 assessments that measured executive function, attention, episodic memory, processing speed, and working memory. These five assessments are explained in detail on the NIHTB website (https://nihtoolbox.force.com/s/article/nih-toolbox-administrators-manual-and-elearning-course). A trained researcher conducted the assessment in a quiet, separate room using an iPad that was placed about one foot away from the seated participant, on a table. Participants were offered a snack (200 kcals, 20 g carbohydrates, 5 g protein) shortly before testing to ensure that hunger had minimal effect on scores. The researcher gave all participants a 5-minute break halfway through the session, which took place after two of the assessments had been completed.

### 2.5. Performance

All performance testing familiarization occurred at visits 1 and 2 to allow the participants to feel comfortable and confident using laboratory equipment when they performed pre-testing. Adaptations were made to standard performance laboratory tests to be dance-specific and are described in detail below.

Performance pre-testing occurred at visit 3 and began with a 5-minute warm-up on a cycle ergometer at a self-selected pace. Following the warm-up, participants were asked to perform isokinetic strength assessments using the Cybex dynamometer: five repetitions of maximal effort hip extension and flexion at 80 degrees per second. Prior to testing, three familiarization trials were completed in each direction and for each limb. To ensure the test was dance-specific, each dancer was asked to perform a battement (kick) forward (hip flexion) and backward (hip extension) from a standing first position (externally rotated). The range of motion limits were set to allow maximal flexion and extension (minus 10 degrees for safety reasons) while the dancer maintained an upright torso posture. The leg that was closest to the dynamometer was the leg being tested. Mean peak torque (Nm) values in each direction and for each limb were used for analysis. Fatigue index (FI) was calculated using the following formula: [(start torque – end torque)/start torque]. A 5-minute break was given following isokinetic testing.

Next, a medicine ball throw was performed to assess upper body power. Participants were instructed to stand behind a line marked on the floor and to throw the medicine ball (6.81 kg) using both hands with fingers pointed in from chest level, similar to a chest pass in basketball, horizontally as far as possible. Participants were further instructed to not use their lower body for power generation and to not step over the line after the medicine ball was released. A tape measure was mounted to the floor, and medicine ball throw was measured in meters. Participants performed three trials; the furthest throw was used for analysis. Rest time between trials was 30 seconds.

Following a 5-minute rest, participants then completed two styles of maximal vertical jumps: feet in parallel and first position (i.e. heels together with toes turned out until heels are in a straight line) (Vertec). To measure jump height, participants stood with their arm in the air and the bottom lane of the vertec was placed at fingertip height. Next, participants jumped as high as possible, striking the vertec lanes forward. Participants performed two practice jumps separated by at least one minute. Following the practice jumps, participants performed three maximal effort vertical jumps for each jump style. The highest jump for each style was used for analysis.

Participants then performed a modified repeated bout Wingate power test to assess anaerobic power and fatigue resistance (approximately 20 minutes following isokinetic testing). Participants performed another warm-up on a cycle ergometer (Monark) at a self-selected pace for 5 minutes to reacclimate to the bike. They then immediately performed two 5-second sprints with a 15-second recovery period in between to complete the warm-up period. Following the warm-up, participants were asked to perform three, 15-second sprints (at maximal effort) on the cycle ergometer with a resistance relative to body mass (0.075*body mass (kg)). Each 15-second sprint was separated by 2 minutes to allow for recovery. The best sprint (highest number of revolutions on the cycle ergometer) was used for analyses. This modified Wingate protocol was created to reflect dance performance which involves short all-out efforts (15 seconds instead of 30 seconds) and minimal recovery (2 minutes instead of 4–6 minutes).

## 3. Statistical analyses

The independent variables in this study were time (pretest and posttest) and group (CR, PL), and the dependent variables were measures of body composition, anaerobic performance, and cognitive measures. A 2-way repeated measures analysis of variance (ANOVA) was used to assess the effect of time*group interactions on each variable (e.g. body mass, LM, FM, mean peak torque). When significant time*group interactions were observed, post-hoc paired t-tests were conducted to determine which groups were significantly different. Descriptive statistics were reported as means±standard deviation. Significance was set at *p* ≤ 0.05. Statistical analyses for all body composition, anaerobic fitness, and dietary intake were conducted using the Statistical Packages for the Social Sciences (SPSS) version 25 (IBM Corp., Somers, NY, USA). Statistical analyses for all cognition outcomes were conducted using SAS® software, version 9.4 (copyright © 2002–2012, SAS Institute Inc., Cary, NC, USA).

## 4. Results

Participant demographic characteristics are reported in Table 1. Thirteen participants (72% of the dance program) completed the 6-week CR supplementation intervention, with seven participants in the treatment group and six participants in the control group. No negative side effects were reported at daily supplementation check-ins, and supplementation compliance was 98%. Additionally, post-hoc observed power analyses revealed a statistical power of 69.6%.

**Table 1.** Participant demographic characteristics (mean ± standard deviation).

	All (*n* = 13)	Placebo (*n* = 6)	CR (*n* = 7)
Age (yrs)	20 ± 1	20 ± 1	21 ± 1
Height (cm)	162 ± 6	163 ± 5	161 ± 7
Body mass (kg)	59 ± 9	59 ± 10	58 ± 10
BMI (kg/m^2^)	22.3 ± 3.0	22.3 ± 3.7	22.5 ± 2.7
Dance training (hrs/wk)	9.4 ± 3.6	11.4 ± 3.5	7.3 ± 2.6
Rehearsal training (hrs/wk)	4.2 ± 5.0	5.6 ± 5.5	2.8 ± 4.4

CR; creatine monohydrate, Yrs; years, cm; centimeters, kg; kilograms, kg/m2; kilograms per meter squared, wk; week.

### 4.1. Body composition outcomes

**Figure 2.**
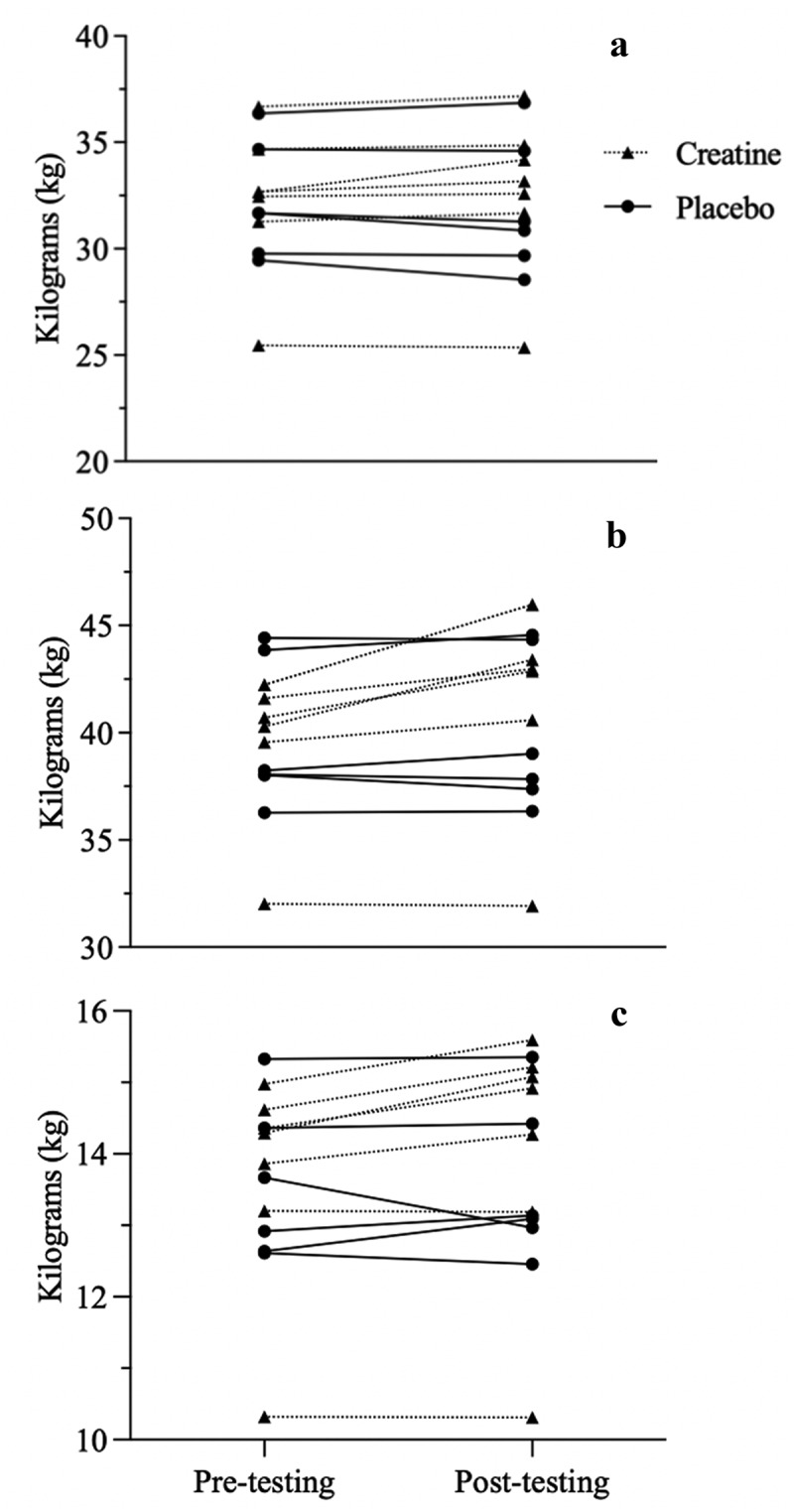
Individual data for total body water (a), lean mass (b), and lower appendicular lean mass (c) from pre- to post-testing Kg=kilograms.

Body composition outcomes are reported in Table 2. Significant time*group interactions were observed for body mass (kg; *p* = 0.010), TBW (kg; *p* = 0.024), LM (kg; *p* = 0.020), and lower body appendicular LM (kg; *p* = 0.047). Individual changes from pre- to post-testing are represented in Figure 2. Simple main effects analysis showed time did have a statistically significant effect on LM (kg; *p* = 0.011, %; *p* = 0.011), FM (kg; *p* < 0.0001, %; *p* < 0.001), upper appendicular FM (kg; *p* = 0.004), lower appendicular FM (kg; *p* < 0.0001) and visceral adipose tissue (VAT) (*p* = 0.002) and appendicular skeletal muscle indices (ASMI) regardless of treatment group. No significant main effects for time were observed for all other measures by group.

**Table 2.** Body composition outcomes.

	Placebo (*n* = 6)		CR (*n* = 7)		
	Pre	Post	Pre	Post	*p-value*
Body mass (kg)	59.4 ± 9.7	57.8 ± 9.0†	58.3 ± 9.8	59.0 ± 10.6	0.010†
Total body water (kg)	32.2 ± 2.7	32.0 ± 3.1	32.2 ± 3.5	32.7 ± 3.6†	0.024†
LM (kg)	39.8 ± 3.4	39.9 ± 3.6	39.8 ± 3.6	41.5 ± 4.5†	0.020†
LM (%)*	67.6 ± 5.4	69.6 ± 5.2	69.1 ± 6.4	71.1 ± 6.2	0.933
Upper Appendicular LM (kg)	3.6 ± 0.2	3.6 ± 0.2	3.7 ± 0.3	3.8 ± 0.4	0.250
Lower Appendicular LM (kg)	13.5 ± 1.0	13.5 ± 1.0	13.6 ± 1.5	14.1 ± 1.5†	0.047†
FM (kg)*	16.9 ± 6.3	15.5 ± 6.0	16.0 ± 7.0	15.0 ± 6.9	0.393
FM (%)*	27.8 ± 5.6	26.2 ± 5.6	26.6 ± 6.7	24.6 ± 7.9	0.506
Upper Appendicular FM (kg)*	1.8 ± 0.6	1.7 ± 0.6	1.7 ± 0.7	1.6 ± 0.6	0.733
Lower Appendicular FM (kg)*	6.8 ± 1.3	6.3 ± 1.1	6.7 ± 2.6	6.2 ± 1.9	0.882
VAT (g)*	257.0 ± 197.7	213.8 ± 188.3	210.8 ± 131.3	176.0 ± 100.6	0.670
ASMI (kg/m^2^)*	6.4 ± 0.3	6.5 ± 0.3	6.7 ± 0.3	6.9 ± 0.4	0.103

†Indicates a significant time*group interaction (*p* ≤ 0.05) by 2-way repeated measures ANOVA.

*Indicates a main effect of time regardless of treatment group (*p* ≤ 0.05) by paired t-tests.

CR; creatine monohydrate, Kg; kilograms, LM; lean mass, %; percent, FM; fat mass, VAT; visceral adipose tissue, g; grams ASMI; appendicular skeletal muscle indices, m^2^; meters squared.

### 4.2. Performance outcomes

Performance outcomes are reported in Table 3. Simple main effects analysis showed that time did have a statistically significant effect on Average Relative peak power output (PPO) (*p* < 0.0001), absolute PPO (*p* < 0.0001), mean power output (*p* = 0.001), and fatigue index (%; *p* = 0.043) regardless of treatment group. Additionally, simple main effects analysis showed that time did have a statistically significant effect on left flexion mean peak torque (*p* = 0.008), and left extension mean peak torque (*p* = 0.027) regardless of treatment group. No significant main effects for time were observed by group (*p* > 0.05).

**Table 3.** Performance outcomes.

	Placebo (*n* = 6)		CR (*n* = 7)		
	Pre	Post	Pre	Post	*p-value*
Avg Relative PPO (W)*	8.4 ± 0.7	9.0 ± 0.7	8.7 ± 0.6	9.3 ± 0.8	0.907
Avg Absolute PPO (W)*	487.1 ± 62.3	520.8 ± 72.1	508.2 ± 76.6	543.9 ± 89.1	0.859
Avg MPO (W)*	426.2 ± 54.4	439.6 ± 58.5	429.2 ± 60.5	453.1 ± 65.8	0.208
Avg FI (%)*	25.4 ± 7.3	29.1 ± 7.5	29.0 ± 8.3	30.2 ± 6.6	0.370
R Flexion Mean Peak Torque (Nm)	57.4 ± 16.1	53.7 ± 11.4	56.8 ± 13.4	47.9 ± 8.3	0.476
R Extension Mean Peak Torque (Nm)	46.6 ± 10.8	39.5 ± 4.2	42.1 ± 9.0	40.6 ± 8.7	0.294
L Flexion Mean Peak Torque (Nm)*	58.5 ± 20.7	48.7 ± 11.7	56.4 ± 10.2	50.4 ± 7.5	0.461
L Extension Mean Peak Torque (Nm)*	40.1 ± 7.1	32.3 ± 4.7	40.2 ± 8.2	36.1 ± 9.4	0.448
Vertical Jump Parallel (cm)	36.2 ± 5.5	37.6 ± 4.2	34.1 ± 6.2	34.6 ± 6.4	0.541
Vertical Jump 1^st^ Position (cm)	32.6 ± 6.2	33.4 ± 3.0	28.1 ± 6.0	29.4 ± 5.3	0.813
Medicine Ball Throw (m)	1.7 ± 0.2	1.8 ± 0.3	1.7 ± 0.4	1.7 ± 0.3	0.758

*Indicates a main effect of time regardless of treatment group (*p* ≤ 0.05) by paired t-tests.

CR; creatine monohydrate, PPO; peak power output, W; watts, MPO; mean power output FI; fatigue index %; percent, R; right, Nm; newton meters, L; left, cm; centimeters, m; meters.

### 4.3. Cognitive outcomes

Cognitive scores are summarized in Table 4. There was not a significant interaction between the effects of time and treatment on any of the cognitive outcome variables. Simple main effects analysis showed that time did have a statistically significant effect on Fluid Cognition Composite score (*p* = 0.02), with post scores significantly higher than pre scores regardless of treatment group. There were no other significant main effects of time or treatment on any other cognitive score.

**Table 4.** Cognitive outcomes.

	Placebo (*n* = 6)		CR (*n* = 7)		
	Pre	Post	Pre	Post	*p-value*
Flanker Inhibitory Control and Attention (CS)	8.3 ± 0.4	8.5 ± 0.5	8.9 ± 0.6	9.0 ± 0.7	0.720
List Sorting Working Memory (RS)	18.5 ± 1.9	19.2 ± 1.0	20.4 ± 2.9	20.1 ± 2.0	0.467
Dimensional Change Card Sort (CS)	8.5 ± 1.0	8.5 ± 0.8	8.6 ± 1.2	8.4 ± 2.0	0.585
Pattern Comparison Processing Speed (CS)	71.8 ± 9.7	73.2 ± 13.3	69.6 ± 19.0	78.4 ± 14.7	0.175
Picture Sequence Memory (CS)	613.6 ± 104.5	657.1 ± 71.3	630.2 ± 95.2	652.1 ± 71.5	0.527
Fluid Cognition Composite (ACS)*	97.5 ± 14.3	102.7 ± 14.3	104.4 ± 24.2	110.6 ± 21.9	0.824

*Indicates a main effect of time regardless of treatment group (*p* ≤ 0.05) by paired t-tests.

CR; creatine monohydrate, CS = computed score, RS = raw score, ACS = age corrected standard score.

### 4.4. Depression, anxiety, and stress outcomes

Depression, anxiety, and stress outcomes are reported in Table 5. No significant main effects for time were observed for depression, anxiety, and stress by group (*p* > 0.05).

**Table 5.** Depression, Anxiety, and Stress Outcomes.

	Placebo (*n* = 6)		CR (*n* = 7)		
	Pre	Post	Pre	Post	*p-value*
Depression	2.8 ± 3.0	2.8 ± 2.6	2.6 ± 2.7	3.7 ± 3.3	0.331
Anxiety	2.0 ± 2.1	2.7 ± 1.6	3.9 ± 3.2	5.2 ± 5.6	0.573
Stress	5.0 ± 2.5	4.5 ± 2.0	9.1 ± 2.3	8.7 ± 4.4	0.969

CR; creatine monohydrate.

### 4.5. Dietary intake outcomes

Dietary intake outcomes are reported in Table 6. No significant main effects for time were observed for all dietary intake outcomes by group (*p* > 0.05).

**Table 6.** Dietary intake outcomes.

	Placebo (*n* = 6)		CR (*n* = 7)		
	Pre	Post	Pre	Post	*p-value*
Calories (kcals)	1740.7 ± 1035.5	1454.7 ± 577.8	1374.1 ± 508.8	1311.4 ± 487.3	0.357
Carbohydrates (g)	195.7 ± 121.0	193.2 ± 94.2	168.3 ± 65.2	171.9 ± 48.7	0.660
Protein (g)	72.6 ± 48.4	49.9 ± 17.2	64.4 ± 21.7	55.7 ± 18.2	0.401
Fat (g)	73.5 ± 40.2	51.5 ± 17.7	51.7 ± 22.9	53.4 ± 18.5	0.102

CR; creatine monohydrate, Kcals; kilocalories, g; grams.

## 5. Discussion

The current study demonstrated significant increases in estimates of TBW and LM following 6 weeks of CR supplementation in female collegiate dancers compared to a placebo control. Specifically, CR supplementation resulted in a significant increase in estimates of lower appendicular LM. Previous literature has established CR supplementation augments LM independent of body mass increases in pre-menopausal females [18–21]. However, the observed changes in DXA LM are likely attributed to increases in TBW due to the inclusion of body fluids in the LM measurements for DXA. In the present study, CR consumption also led to an increase in body mass gain as demonstrated by significant increases in total body mass. These results may elicit apprehension in the collegiate dance population due to a heightened focus on body image and body mass as a function of this aesthetic sport. Additionally, data from the present study suggest collegiate dancers’ FM (kg and %) and visceral adipose tissue significantly decreased over time, independent of CR supplementation. These data indicate collegiate dancers lose FM, specifically as abdominal adiposity surrounding the viscera, over the course of 6 weeks during the academic year. These findings are important because it is well documented in previous literature that excessive abdominal adiposity is detrimental to overall health [22]. However, previous data in a collegiate dancer population suggest body composition does not change over the course of one semester or up to two academic years when dietary and exercise interventions are not administered [12–14,16]. Therefore, increases in LM, albeit from increases in TBW, in conjunction with FM decreases over the semester may be appealing for the collegiate dance population whose primary focus is aesthetics. Furthermore, CR supplementation is a safe, easy to administer, minimally invasive strategy to optimize body composition for a collegiate dance population who has little time due to balancing academic and performance demands. However, to the authors’ knowledge this is the first study to investigate CR supplementation in addition to dance training (independent of a resistance training) and further research is needed in a female collegiate dancer population.

In the present study, no significant improvements in performance were observed. This may be explained by the shorter length (6 weeks) of the intervention or absence of resistance training program implemented in the present study. Additionally, although familiarization sessions were performed, these maximal effort laboratory tests are unfamiliar to the dance population and may have contributed to large standard deviations in performance measures. However, previous literature suggests CR supplementation improves lower-body muscular strength and power in untrained females following 10 weeks of CR supplementation (20 g·day **−1**) independent of resistance training [23]. Supplementation with CR is typically employed using a loading phase (0.3 g·kg **−1** ·day **−1** for 5–7 days) strategy to fully saturate CR monohydrate stores followed by a maintenance phase (0.03 g·kg**−1** ·day **−1** for >3 weeks) [2]. In the present study, a loading phase was not employed due to the collegiate dance population’s heightened focus on body mass and aesthetic appearance. Had a loading phase been employed, significant increases in total body mass may have been exacerbated, increasing apprehension among this population. However, previous research suggests a CR loading phase may be necessary to elicit changes in muscular strength and power [21]. Furthermore, average relative peak power output, absolute power output, and mean power output significantly increased over time independent of the treatment group. These data suggest collegiate dancers’ anaerobic fitness does improve as a result of the dance program alone across 6 weeks of the semester. Interestingly, left flexion mean peak torque, left flexion power, left extension mean peak torque, and left extension mean power significantly decreased over time independent of the treatment group. These results may be explained by differences in limb utilization during dance training, with an increased emphasis placed on the non-dominant limb. However, the researchers were surprised by these results, and further exploration of limb dominance and lower limb muscular strength warrants additional investigation.

There is previous literature that supports the use of CR supplementation to improve certain cognitive functions, such as short-term memory [24–26]. However, CR supplementation studies show inconclusive results in cognitive functions such as inhibitory control, processing speed [27,28] executive function, and memory [25,29], indicating a need for further research on the effect of CR supplementation. This study’s 6-week supplementation of 0.1 g·kg **−1** ·day **−1** did not influence any of the cognitive measurements tested by the NIHTB in collegiate dancers. This could be due to a few factors within this study, such as participant activity level, lack of heterogeneity, small sample size, or age. The significant effect of time on the Fluid Cognition Composite score that was found in this study would suggest weakness in the test-retest validity of these scores or a participant learning effect, but this result is more likely due to study limitations, as previous literature has found that the NIHTB Cognitive Battery has high test-retest validity among adults [30,31]. Although the results of this study did not support researcher expectations that CR would improve cognitive functions, more research should be done on a larger sample size and more heterogeneous participant pool in order to better understand the effect of CR on collegiate dancers.

Collegiate dance is unique, due to the large mental and physical demands brought about by rigorous academic schedules as well as a lack of nutrition and supplemental training resources. Previous data suggest CR supplementation augments brain CR via the blood-brain barrier, thereby eliciting an inverse relationship with depressive symptoms [9,10]. Furthermore, in individuals with methamphetamine dependence, 8 weeks of CR supplementation significantly decreased anxiety symptoms [11]. However, data from the present study demonstrate no changes in depression or anxiety following 6 weeks of CR supplementation, albeit interesting findings pertaining to stress. The Depression, Anxiety and Stress scale was utilized to capture participants’ mental health during the intervention. These data indicate stress was significantly increased over time for the control group while stress was significantly decreased for the CR group, albeit independent of the treatment. Therefore, there may have been an inadequate sample size to detect a group difference in stress.

Strengths of the study include high compliance due to researchers’ access to collegiate dancers within the University of Idaho department, and the use of the NIH Toolbox. The NIH Toolbox is a widely used and validated method of measuring various cognitive functions and will allow easy comparison of scores across other studies that have used the same tool. The study was randomized and placebo-controlled, and covered a large variety of physical movements, making it thorough in physical assessments. Additionally, dietary intake was used as a control to ensure participants’ habitual dietary intake was not influenced by the intervention. No significant differences in dietary intake were observed by time or group. These data indicate collegiate dancers’ dietary intake did not change over the course of the intervention.

There were a number of limitations that may have affected the outcome of the study. The study had a high level of homogeneity and relatively small sample size (*n* = 13) contributing to the low statistical power of the present study. Yet, there is a small body of evidence suggesting creatine supplementation alone, independent of resistance training, may elicit health benefits [3]. All dancers were female, within a small age range, had a similar educational background, and resided in the same geographical location. Additionally, participants were instructed to maintain habitual dietary intake throughout the study; however, it should be noted we did not perform 24-hour dietary recalls to assess dietary intake leading up to pre- and post-testing. Although hydration instructions were provided to participants, urine specific gravity was not assessed prior to measuring TBW using InBody. Dancers in the placebo group were cast in more dance performances for the fall show (auditions were in week 2 of the study) and therefore rehearsal hours were higher than the CR group. However, rehearsal hours are notoriously low intensity (10.17 ± 6.63 ml/kg/min [32], and it is unlikely that this contributed to group differences. No measures of muscle mass (i.e. MRI cross-sectional area) were obtained in this study, so changes in LM cannot be attributed to changes in muscle mass. Lastly, due to the nature of dance training, lab performance testing may have required more familiarization sessions to understand maximal effort.

Data from the present study demonstrate CR supplementation is an effective means to increase LM in a collegiate dance population independent of a resistance training program. These data indicate a novel approach to optimizing body composition with a safe, noninvasive technique which can be employed in a population with rigorous academic and training demands. Further investigation is necessary to determine if CR supplementation at various doses and timeframes may elicit increases in LM as well as performance capabilities in female collegiate dancers.
